# History of Crowdfunding in the Context of Ever-Changing Modern Financial Markets

**DOI:** 10.1007/978-3-030-46309-0_10

**Published:** 2020-04-25

**Authors:** Aki Kallio, Lasse Vuola

**Affiliations:** 1 School of Business and Law, University of Agder, Kristiansand, Norway; 2 School of Business and Law, University of Agder, Kristiansand, Norway; 3 School of Business and Law, University of Agder, Kristiansand, Norway; 4 Danske Bank A/S Finland Branch, Helsinki, Finland; 5 Fundu Ltd., Helsinki, Finland

**Keywords:** Financial markets, Financial innovation, Financial technology, Financial history, Opportunism, Moral hazard, Financial regulation, Financial system

## Abstract

The history of financial markets and finance are united by continuous fluctuations between economic cycles usually caused by structures that enable opportunism and moral hazards. Every crisis contains the seeds of change and innovation, but also risks for regulative overreactions. Crowdfunding as a form of financing is part of this series of innovations in the history of the financial markets. Understanding of the historical changes of both the financial market and the financial system as a whole helps to put new financial innovations, such as crowdfunding and, more broadly, fintech into perspective. The evolution of financial markets or corporate finance naturally will not end in crowdfunding. This chapter gives an overview of history of crowdfunding as part of the ever-changing modern financial markets and contextualizes it as a new, innovative, and modern form of financing in the financial markets.

## Introduction

Crowdfunding is a means of raising finance for projects from the crowd often through an internet-based platform where project owners pitch their idea to potential backers, who are typically not professional investors, although increasing activity by institutional investors has been recorded. Crowdfunding takes many forms and sometimes without any potential for a financial return. Crowdfunding in its current context is relatively young and business models are evolving at a fast pace. Crowdfunding platforms have emerged recently since internet technology evolved in such a way as to allow easy and simple two-way communication. This enables interaction between the members of the crowd of investors, as well as between the crowd and the project owners pitching their specific projects (European Securities and Markets Authority 2014).

In 2017, alternative finance volumes from across Europe grew by 36%, from 7.67 billion euros to 10.44 billion euros (Ziegler et al. 2019). Overall, the major share of European volume still originates from the UK (68%). However, excluding the UK, the European online alternative finance market grew at nearly double the UK’s year-on-year growth rate—63% in comparison to 35% in 2017. While this growth was not as strong as in 2016 (101%), there was visible growth in each sub-region of continental Europe. As a whole, the market grew by just over 1.3 billion euros to 3.369 billion euros in 2017 (Ziegler et al. 2019). While there was an overall growth, the rate of growth seems to have cooled in some more mature markets even though it is continuing (Ziegler et al. 2019).

The growth of crowdfunding as a new complementary and alternative form of financing is indisputable, and its importance to businesses both domestically and internationally is already remarkable (Ziegler et al. 2019). With the ever-increasing advent of digitalization combined with tightening regulation for banks, alternative finance has become an important part of the present financial markets. The alternative of today may turn out to be the mainstream of tomorrow. It is therefore important to evaluate the role of crowdfunding as part of the history of the financial markets. This is particularly relevant in the context of what is currently happening in financial markets via the transformation brought about by digitalization and ever-increasing regulatory burden imposed especially towards banks while restricting their ability to finance small and medium-sized enterprises (SMEs). Crowdfunding, at least for the time being, can be considered one of the most viable examples of the gradual transformation of financial markets caused by emergence of financial technology (fintech). Thus, crowdfunding joins an important group of innovations, which have changed, formed, and developed the financial markets through time like credit cards, stocks, mutual funds, and online banking, all of which have been influential innovations and disruptors of their time (Atack and Neal 2009).

However, as a phenomenon, there is nothing new in crowdfunding and similar ways to access finance have been utilized in the past. Currently crowdfunding is increasingly establishing itself as an integral part of the finance industry especially for start-ups and early phase companies that have traditionally been funded by “family, friends, and fools” in order to develop and gradually attract more interest (by direct investments and/or other collaboration) from sophisticated investors and venture capital funds (Kallio and Vuola 2018; Ziegler et al. 2019). This chapter focuses on those forms of crowdfunding, which have the most relevance to the financial markets, namely debt- and investment-based crowdfunding, and aims to give an analysis why, what, and how crowdfunding has become the phenomenon we are witnessing today and at the same time contextualize it as one of the continuous innovations in the history of ever-changing modern financial markets.

## Fundamentals of the Financial System

The financial system is a complex environment comprising of different markets that use various financial instruments, such as equities and bond markets, and includes a number of different institutions such as pension funds, banks, insurance companies, funds, large companies, and retail investors (Drake and Fabozzi 2010). The purpose and functioning of financial markets from an economical perspective is based on a fairly simple point of view: markets channel money from surplus sectors to deficit sectors. This mechanism leads, in theory, to the allocation of capital in a most efficient and profitable way for the economy as a whole. Well-functioning financial markets and financial system in general are a prerequisite for the economic activity and growth we are familiar with. In market driven economies, general welfare is strongly connected to efficiency of the markets (Drake and Fabozzi 2010; Kallio and Vuola 2018).

Main functions of the financial markets are (i) providing information to and between market participants, which at best makes the market work transparently and allows the information available to be immediately, equally, and correctly reflected in the prices of the financial instruments; (ii) enabling efficient allocation of funds from the surplus sector to the deficit sector often through intermediaries such as banks so that funding for necessary investments can be acquired at the lowest possible cost and without delay; (iii) risk management aimed at proportionate spread of the risk inherently built into financial markets to be divided among different investments quantitatively over time; and (iv) providing liquidity, the purpose of which is to enable an investment acquired from the financial markets to be cheaply, easily, and quickly liquidated to cash (Atack and Neal 2009; Drake and Fabozzi 2010).

The usual cause of acquiring financing is rooted in a situation where a company’s (or private person’s) own capital is not sufficient to carry out the necessary or targeted investments, cover running costs, or overcome unexpected costs. In these cases, equity or debt financing become the most viable option. Financing is a way to mobilize resources quicker compared to collection of such resources by cash flow, which would take a considerable amount of time. The leverage function of new capital enables faster growth, but it involves a cost. In practice, the company is always forced to pay compensation for the use of the capital it has acquired. Equity financing is in practice direct equity investments into the company in which the investor receives an ownership share equalling the value of his investment in the company. The return on equity investment consists of the profit distributed by the company as well as profits re-invested into the company. These may increase at par if the valuation of the holding in the company increases, so the return on equity investment is theoretically unlimited. Similarly, the risk is at most equal to the invested equity (not more, not less) (Ferran 2008; Drake and Fabozzi 2010).

Debt financing is both short term (i.e. for a period of less than one year), such as trade payables and overdrafts, and long term (i.e. over a period of one year or more), such as bonds and bank loans. Debt financing is always external financing, and, as such, there is always an underlined obligation to repay on fixed terms compared to equity. On the other hand, debtor also carries the credit risk and the risk of the company having sufficient cash flow, which the lender must carefully consider when making a financing decision in addition with the evaluation of potential collaterals. The risk of an unsecured debt investment is at most equal to the borrowed capital and overdue, accrued and unpaid interest related thereto (Ferran 2008). Often an investor seeks to secure his position contractually, but also by using various collateral arrangements that secure status of the creditor in the event of a serious default and ultimately in the event of insolvency of the company (Ferran 2008). Debt financing takes precedence in the ranking of the payment order in case of an insolvency of the company compared to equity financing (Ferran 2008). Since profit is a reward for risk taken in business, the lower rank of equity means more risk compared to debt. Therefore, the profit expectancy in equity is generally higher than in debt (Drake and Fabozzi 2010).

The board and the management of a company have a fiduciary duty towards the owners. Rational companies aim to optimize their financing seeking for the best available capital structure. With debt financing the company can, from the owners’ perspective, often lower the total cost of capital because investors usually require higher risk premium in relation to equity investments than for debt investments (Drake and Fabozzi 2010). In general, owners of the company try to protect themselves against dilution of ownership making debt finance often a lucrative way to grow through leverage (Ferran 2008). In addition, liability to pay interest in relation to debt financing might provide opportunities to optimize corporate taxation of the company in some jurisdictions (Drake and Fabozzi 2010). With the current stagnating low interest rate era, high leverage ratio may well seem lucrative from owners’ perspective.

## Setting the Scene

The history of financial markets and finance are united by continuous fluctuations between economic cycles from bull markets to bear markets or bubbles to recessions as well as crises usually caused by structures that enable opportunism and moral gambling. Every crisis contains the seeds of a change, but also risks for regulative overreactions, as well as drastic market reactions. One example is the Great Depression of the US in 1929, which was caused by virtually free speculative trading of stocks and derivatives to the general public and the loss of trust through separation of ownership, as explained by Berle & Means in *The Modern Corporation and Private Property* in 1932. Another and more modern example is the subprime crisis that began in 2007, which was caused by securitization of speculative mortgages and secondary markets related thereto, which at first stage caused widespread credibility gap between banks (i.e. credit crunch), and then later spread across the financial markets as a whole. This latter crisis gradually grew into a worldwide financial crisis eventually leading to the European sovereign debt crisis when several European countries experienced the collapse of major financial institutions, bankrupts of numerous of the countries’ biggest companies, high government debt, and rapidly rising bond yield spreads in government securities (Bradley 2013; Chambers and Dimson 2016).

The European sovereign debt crisis also heavily influenced later changes to functioning of and initiatives taken by the European Central Bank (ECB) such as (i) the long-term refinancing operation (LTRO), which is an enhanced credit support measure to support bank lending and liquidity in the euro area announced in 2011, (ii) the targeted longer-term refinancing operations (TLTROs), which are euro system operations that provide financing to credit institutions announced 2014, 2016, and 2019, respectively, and (iii) the asset purchase programme (APP), which is part of a package of non-standard monetary policy measures that also includes targeted longer-term refinancing operations initiated in mid-2014 including corporate sector purchase programme (CSPP), public sector purchase programme (PSPP), asset-backed securities purchase programme (ABSPP), and third covered bond purchase programme (CBPP3). The aim of the ECB with abovementioned programmes was on the one hand to offer banks long-term funding at attractive conditions in order to preserve favourable borrowing conditions for banks and stimulate bank lending to the real economy and on the other to support the monetary policy transmission mechanism and provide the amount of policy accommodation needed to ensure price stability (European Central Bank 2020). In addition, the crisis acted as a catalyst to a still persisting zero-level (or even negative) interest rate environment in Europe.

The former (i.e. Great Depression) led to the implementation of two important acts in the US. First, the Banking Act (i.e. the Glass–Steagall Act), which prohibited any one bank from both accepting deposits and underwriting securities, in order to ensure that if a bank made significant losses underwriting securities, deposits would not be adversely affected. And, second, the extremely tight Securities Act of 1933, representing the first major federal legislation to regulate the offer and sale of securities in the US in order to ensure that buyers of securities receive complete and accurate information before they invest in securities, which is still in force in the US with only some relief from the original statute (Cassis 2017; Mitchener 2005). Both Acts restricted banks’ business opportunities largely for the benefit of the general public and society as a whole.

The latter caused tightening of bank regulation, such as risk-weighted capital requirements, market condition, and investor protection, in the global financial markets (especially in the US and Europe) (Chambers and Dimson 2016, pp. 193–194). The enactment of the Dodd–Frank Act in the US was a response to the subprime crisis and brought about the most significant changes to financial regulation in the US since the 1930s preventing the US government from bailing out failing banks with taxpayers’ money and imposing short-selling restrictions. In Europe, similar legislative changes were implemented and, with enactments of, among others, the Capital Requirements Directive IV (CRD IV) and the Markets in Financial Instruments Directive II (MIFID II), many restrictions were imposed on banks’ businesses. Actions taken both in the US and Europe have heavily impaired banks’ business opportunities, by way of, among others, tying their capital to much higher ratios than before the crisis, preventing or even restricting the use and leverage of their balance sheets as well as increasing regulatory compliance and wider conduct requirements (Zestos 2016).

This restrictive trend, as described, has been particularly strong in Europe, with the result that especially the financing of small and medium-sized enterprises (SMEs) has become more challenging. This has been counterbalanced by large-scale EU-wide financing and guarantee arrangements, whose long-term effects are still unknown. In future, we shall learn whether this partial “socialization” of credit risk to the taxpayers was an effective means to counterbalance the tightening regulation. Examples of these approaches, include a corporate bond purchase programmes started by the ECB (as referred above) and the setting up the European Fund for Strategic Investments, which is an EU budget guarantee that provides a shield for the European Investment Bank covering most risky part of the projects it has funded. In authors’ view, once these instruments have been introduced to the markets, it may be hard to withdraw them even in the bull market leading into a long-term partial socialization of SME credit risk to taxpayers.

Like other forms of financing, crowdfunding always works within a particular jurisdiction. The provisions laid down in the regulation, in particular the mandatory ones, must be taken into account when utilizing all forms of financing. Besides understanding the history and functioning of global financial markets, it is always necessary to place the activity within the given operating environment and regulations related thereto (Drake and Fabozzi 2010). At the same time social institutions, such as governments, central banks, market supervisors, and supranational institutions, strive to promote trading to maintain economic growth while contrary to this goal also control the markets and operations therein in order to prevent the emergence and spread of systemic risks. Financial law includes acts, which in many cases point to opposite ways aiming at enabling efficient exchange to support investment, economic growth, and employment, and, at the same time, to prevent actions threatening the basic operation of national economies through avoiding emergence of systemic crises. The goal of financial market legislation is simple: trying to optimize the functioning of the financial market. Efficiency in the financial markets does not mean extreme liberalism. On the contrary, the financial market regulation should be limited to what is necessary so that overall confidence in the financial system remains (Drake and Fabozzi 2010).

Every statute increases complexity of the legal system in a non-linear manner. New regulation may lead to artificial market practices and efficiency losses for all market players. Hence, regulation should, from a market liberal economic perspective, focus on ensuring the functioning of key market mechanisms with minimal interruption. In *Confusion de Confusiones* Joseph de la Vega well stated in 1688 that financial system is at the same time “the fairest and most deceitful business … the noblest and the most infamous in the world, the finest and most vulgar on earth”. Things have not changed so much after de la Vega. The aim for the regulator is to incentivize the fairness and nobleness and de-incentivize the deceitfulness and vulgarness.

Efforts to maximize the interests of different stakeholders in the financial markets, and competition among them, create incentives for moral gambling, which lawmakers seek to counter by creating and imposing counter-incentives as well as effective control and enforcement systems. Financial market regulation always affects competitiveness of stakeholders in the financial markets, and regulation that is too burdensome can be seen detrimental to the whole financial market system. On the other hand, legislation can also help speed up market disruption (PWC 2017). Delays are a challenge for the legislator: decision delay, legislative delay, and implementation delay cause problems for effective and well-functioning legislation. The longer the delays the legislator is facing are, the easier it is for crises to emerge and the deeper they can become.

Similarly, the faster the new forms of financing, innovations, and practices are emerging in the financial markets, the more challenging is the role of the financial market supervisor and the legislator. However, as the legislator and market supervisor seek to control systemic risk by observing and regulating existing phenomena, new forms or models and other financial innovations are evolving at an ever-increasing pace in the financial markets. Of these, crowdfunding is an illustrative example. A considerable amount of new financial regulation has come into effect during the last years affecting those operating in the financial markets by increased costs and complexity. This emphasises the ongoing struggle between the stakeholders operating in the financial markets and the broad, ever-increasing, and multi-level regulation shaping the fundaments of financial ecosystem (Kallio and Vuola 2018).

## The Brief History of the Modern Financial System

The development of the international financial system is in every respect a historical, economic, and political process. Because of this, it is essential to briefly outline the past, in addition to the present, in order to be able to assess potential future developments and guidelines of the financial markets. The beginning of the international financial system as we know it today dates back to the 1970s, but, more broadly speaking, a global financial system has existed much longer. This further stresses the importance to understand events, notions and wider developments described in the written financial history, which provides the means to comprehend functioning of modern financial markets. In *On the Genealogy of Morals* Friedrich Nietzsche noted in 1887 that the whole idea of duty and personal obligation is rooted in the oldest and the most primitive relationship there is, the relationship between creditor and debtor. This statement continues to quite accurately describe fundamental relationships in the modern financial markets.

The financial markets tend to operate in cycles, which differ depending on the subject matter (volatility, share prices, etc.) under consideration. For example, it is possible to assess the business cycle or the stock market cycle, which largely differ from each other due to differences in relation to the underlying subject matter in question. Although history may not be said to repeat itself, the cyclicity of the financial markets has largely been scientifically proven (Marmer 2016; Chambers and Dimson 2016; Atack and Neal 2009) although the timing of different cycles cannot be determined with any precision.^1^ Therefore, it is not surprising that the financial markets witness both highs and lows, of which the former can in the worst case create a financial bubble^2^ and the latter a recession meaning a deeper and longer lasting economic downturn.^3^ Previous major changes in the financial markets may be categorized in many ways (Atack and Neal 2009). They can be approached through economic bubbles in relation to their impact on the real economy.

One way to outline the most important financial market development stages is to divide them into five phases. In the first phase in the nineteenth century, the leading European industrialized countries and the colonized non-European regions they ruled moved to a gold-denominated currency system that collapsed during World War I. There were sincere efforts to return the gold-denominated currency system in the 1920s, but they failed. This can be considered the second phase of the financial markets’ development. In the third phase, the Great Depression of the US, followed by significant tightening of the US financial market regulation and eventually World War II caused international financial markets to shut down almost completely. The fourth stage of the international financial system began after World War II based on gradual dismantling of the post-war regulated economy and opening of the international financial markets, which lasted up to the oil crisis of the 1970s. After the 1970s, we have more or less lived in the current historical era comprising building of the global financial system based on neoclassical theoretical approach and characterized by ever-increasing globalization. This can be called the fifth stage, which we are still in (Kari 2016). The end of the 2010s has been marked by a certain degree of inward turning tendency, during which even many influential parties have openly denied functioning of the open and global financial system. The future will show whether we are in the middle of changing paradigm and living the beginning of the new sixth stage in which the international financial system is being gradually overtaken by separate national and inward-looking systems such as we are currently, at least to some degree, witnessing in the US, Brazil, and Russia to a greater extent. Recent outbreak of COVID-19 virus might further accelerate such inward-looking tendencies on a global level.

Understanding of historical changes of both the financial market and the financial system as a whole will help to put new financial innovations, such as crowdfunding and, more broadly, fintech into perspective (Chambers and Dimson 2016). The change in the financial markets is an extremely wide and complex matter influenced by technological advances and digitalization. Also, the current political, economic, and ideological conditions affect the financial system as a whole. While international development seems to be moving towards an increasingly global financial market (despite some inward-looking tendencies), diverse corporate cultures, differences in politics, as well as legislation between countries remain prevalent.

## The Modern Emergence of Crowdfunding

There is nothing new in sourcing money from the crowds. However, crowdfunding, as a concept, is a modern financial service enabled by advanced digitalization. The underlying technology of which has the potential to help investors to find ventures and projects, which need financing and, accordingly, allows the ventures or projects to find investors and backers to finance their growth and development (Dresner 2014, p. 3). Based on one definition “crowdfunding” refers to the ability of pooling small amounts of capital from a potentially large pool of interested funders and supporters (Short et al. 2017). This definition, however, is close to the definition of an initial public offering (IPO). The ability of pooling in IPO is mainly based on the marketing efforts of investment banks acting as “underwriters” whereas in crowdfunding it is based on the digital online platform and its functionalities. In recent years, we have witnessed hybrid models where IPOs have also been executed through crowdfunding platforms.

A crowdfunding platform is “an internet application bringing together project owners and their potential backers, as well as facilitating exchanges between them, according to a variety of business models” (Shneor and Flåten 2015, p. 188). The crowdfunding platforms act as intermediaries between investors and companies (or other projects) and facilitate opportunities for investors to find and support the projects they are interested in (Spacetec 2014). The platform’s core value proposition is in taking down the transaction costs and lowering the bar to start a fundraising campaign effort. Just a decade ago, it was basically impossible for an early stage venture to reach out to tens of thousands of potential investors in a cost-efficient way.

Thus far, crowdfunding has been gaining ground very rapidly (European Commission 2016). Major contributing factors to this growth and spread of crowdfunding are both the international crisis in the financial markets in 2008 that has led, inter alia, tightening the capital adequacy and solvency requirements for credit institutions, and the explosion of internet usage and usability, which together have made it possible to reach large crowds of potential funders in a cost-effective manner (IOSCO 2015).

In the near future, crowdfunding may become an increasingly important source of non-bank financing. Worldwide crowdfunding market has been estimated to reach 371 billion euros in 2017 and based on market data strong growth in recent years has been continued (Ziegler et al. 2019), although the rate of growth seems to have cooled in some more mature markets (Ziegler et al. 2019). Crowdfunding is increasingly establishing itself as an integral part of the finance industry especially for start-ups and early phase companies that have traditionally been funded by “family, friends, and fools”. Furthermore, crowdfunding provides a feasible alternative to unsecured bank loans that have, for the time being, been one of the most important sources of external financing for SMEs in some jurisdictions, while being almost non-existent in others (European Commission 2018b).

## A Brief History of Crowdfunding

Crowdfunding as a form of financing is not a new phenomenon (Spacetec 2014). A similar approach has been used to manage investment risks before internet time (Dresner 2014). For example, in shipping, one of the oldest forms of risk management are guarantee agreements between traders and shipping companies, in which upon the event of loss of cargo all pay part of such loss, but when cargo arrives safely, all parties to the contract (i.e. the guarantors) will receive their proportional share of the profits. This approach has provided the necessary financing to carry out high-risk projects and at the same time enabled successful diversification of the risk associated with the project between the parties.

The basic principles of the crowdfunding business go back to the early eighteenth-century Ireland, where “forefather of microcredits” Jonathan Swift^4^ founded the Irish Loan Fund. The Fund offered small loans to low-income rural families who did not have the collateral required by large banks or proper credit history. By the nineteenth century, more than 300 schemes were implemented in Ireland in all of which small amounts were lent by private investors to individuals who needed a loan for short periods.

One of the early contemporary crowdfunding campaigns was carried out in the US in 1885 when the project of the Statue of Liberty on Liberty Island off New York had run into severe financial difficulties. When other means had proven ineffective, Joseph Pulitzer decided to launch a fundraising campaign to fund the erection of a pedestal for the Statue of Liberty in his own newspaper, *The New York World*. In exchange for a donation, he promised to publish the names of all donors in his magazine regardless of the amount. Over 160,000 donors in about five months had donated more than US $100,000 to erect the pedestal. Most of the donations were quite small—from a few cents to one dollar (Dresner 2014). However, while possibly the most famous and most often cited, the Statue of Liberty project was not the first crowdfunding campaign.

Even earlier examples of crowdfunding are evident. One example is when poet Alexander Pope set out to translate Greek poetry into English in 1713, an effort that included the translation of Homer’s epic poem, “The Iliad”, and asked donors to pledge two gold guineas to support his work in exchange for having the donors’ names published in the acknowledgements of an early edition of the book. Another example is that in the end of the eighteenth century, the famous composer Mozart took a similar path. He wanted to perform three piano concertos in a concert hall in Vienna and published an invitation to prospective backers offering manuscripts to those who agreed to donate funds for this purpose. This approach mirrors the way in which Kickstarter operates today, where campaigners offer backers the first chance to get access to new products offered in campaigns. However, while Mozart failed to reach his funding goal on his first attempt, he succeeded a year later in a second attempt, where 176 backers donated enough funds to bring his concerto tour alive and they were all mentioned in his concertos’ manuscript.

Muhammad Yunus further developed Jonathan Swift’s idea on microcredits and microfinance by founding the Grameen Bank in 1976 (being authorized in 1983 by national legislation to operate as an independent bank in Bangladesh). The goal was to grant loans for entrepreneurs too poor to qualify for traditional bank loans. The bank’s funding has come from different sources, and the main contributors have shifted during times from bulk agencies to central bank of Bangladesh. Grameen Bank is founded on the principle that loans are better than charity to interrupt poverty: they offer people the opportunity to take initiatives in business or agriculture, which provide earnings and enable them to pay off the debt and start a social climb. The Bank has offered credit to classes of people formerly outscoped: the poor, women, illiterate, and unemployed people. Access to credit is based on reasonable terms, such as the group lending system and weekly instalment payments, with reasonably long terms of loans, enabling the poor to build on their existing skills to earn better income in each cycle of loans. He and Grameen Bank were jointly awarded the Nobel Peace Prize in 2006 for their efforts through microcredit to create economic and social development from below (Grameen Bank 2006).

Between 1996 and 1997, the British rock band Marillion funded its tour in the US by collecting US $60,000 from its fans via the internet. This project and other successful fan-based funding rounds that followed gave a boost to the increasing popularity of contemporary crowdfunding from the beginning of twenty-first century. Wider utilization of the form of financing and the spread thereof was made possible by the ever-increasing accessibility to the internet and its growing use by both businesses and households, which in turn made it possible to cost-effectively reach a large crowd at the same time. ArtistShare was one of the first modern crowdfunding services when it was released in the US in 2003. Through its service the artists had, and still have, the opportunity to seek funding to cover their recording costs from a wide audience such as their own supporters and fans . Here, supporters making financial contributions receive the right to download the artist’s album (or song) once it is completed. The success of ArtistShare has also attracted other players to the market, of which perhaps the best known and most successful are reward-based platforms Indiegogo since 2008 and Kickstarter from 2009.

**Fig. 10.1:**
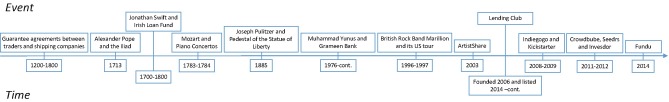
Timeline—Brief history of crowdfunding

When donation and reward-based crowdfunding started to become widespread successes, it was relatively clear that a similar approach would also be used in the capital markets to raise investment-oriented finance. During the last decade, the market started to see platforms seeking to enable capital raising from investors by utilizing opportunities offered by the internet to collect and share investment information in an easier and faster manner, while simplifying the process and using standard terms. Here, the goal was to simplify, to the extent possible, the acquisition of finance from previously heavy and burdensome processes by using modern technology. In the past, acquisition of finance from angel investors lasted at least a number of months, but by using the internet the same funding could be secured within days or at most within a few weeks. One of the most successful pioneers in the industry are the US-based peer-to-peer and business-to-business lending platform—Lending Club, founded in 2006 in San Francisco and listed in December 2014 on the New York Stock Exchange (Freedman and Nutting 2015), and UK-based Zopa, which was launched in 2005 (Kupp and Anderson 2007) as well as Finnish-based equity platform Invesdor, which was founded in 2012, and being the first MIFID II licensed crowdfunding platform to operate cross-border in Europe (Fig. 10.1).

## Crowdfunding and Its Significance in the Modern Era

Starting in 2007 from the overheated housing market in the US, and reaching full speed in 2008, the financial crisis has significantly changed the functioning of international financial markets. Increased regulation, and in particular the tightening of capital requirements for banks, has contributed to the need to find new sources of finance for businesses. Tighter regulation, especially the risk-weighted capital requirements has limited the number of companies that banks could provide debt finance for, and, in turn, led to increased borrowing costs. Therefore, it can be argued that at least to a certain extent, the changes described here have reduced the capacity of credit institutions to meet the financing needs of companies. In addition, a weak and precarious economic situation, which has only recently turned for better, has increased the risk of credit losses and thereby reduced banks’ risk appetite (European Commission 2013). The situation has had a particularly strong impact on European SMEs, which have, due to historical reasons, been dependent on bank financing and, hence, resorted to alternative sources of financing (European Commission 2013, 2015). This is expected to affect the diversification of financial markets in the future. In particular, changes in the regulation of financial institutions can be seen as a limiting factor on the effective functioning of financial markets for entrepreneurial finance and growth companies.

The financial position and access to finance for growth companies and SMEs have weakened to some extent globally. As a result, companies have not always been able to meet the funding needs for their projects from existing sources of finance, which has in certain situations led to a financing gap (European Commission 2018b). Of course, not all the companies are fundable by any means of finance. However, statistics published by European Central Bank have shown evidence of a decline in access to finance for growth companies and SMEs especially in Europe in the aftermath of financial crisis even though situation has gradually improved in recent years. Based on surveys, also covenants (i.e. special conditions) as well as the security requirements of corporate loans have been tightened and interest for corporates, but especially SMEs risen (European Central Bank 2019). Although the situation in Europe is relatively good in comparison to other continents, it has developed for the worse since the financial crisis. Structural deficiencies, overcapacity, low/negative interest rates, and the absence of a pan-European banking regulatory agency have all likely contributed to European banks experiencing persistent profitability challenges (Deloitte 2019).^5^ In Europe, the proportion of SMEs that mention access to finance as one of their main problems, and hence feel that they are not able to drive all potentially profitable projects, has grown (European Central Bank 2019). These findings may well be proof that the EU and national level SME guarantee facilities have not had the expected outcome. In addition, it is uncertain how socialization of credit risk affects the economy as a whole in zero (or even negative) central bank interest environment.

Low interest rates weaken banks’ profitability and reduce the transparency of the actual price paid by the customer, which depends on not only each customer’s financial status and profitability of the business but also the banks’ current fundraising costs and the pursued level of profitability. Based on business and investor surveys (such as European Central Bank 2019) growth companies experience slower growth and higher growth thresholds. Hence, businesses, as they continue to grow, are often lacking access to finance and ability to entice new owners through listings. Low availability of alternative sources of funding, lack of expertise, and the relatively high cost of the listing process, as well as negative attitudes of owners, slow down the growth path of companies.

Therefore, from the perspective of growth-oriented companies, funding opportunities that are complementary or alternative to bank financing, such as risk and equity finance (i.e. bond markets, crowdfunding, venture capital) have increased their importance. Equity crowdfunding is especially important to finance the growth of technology-intensive businesses and innovative companies in general. This is even more relevant when a company is looking for new markets or planning to develop new products. Palmer has concluded in his study that the price of the (crowd)funding (i.e. associated costs) is not the main reason why some companies decide to use crowdfunding instead of traditional sources of finance. The main reason for companies to avoid bank-based financing is, according to Palmer, to avoid the heavy bureaucracy involved in dealing with banks in the first place (Palmer 2016).

The prevailing (zero or even negative interest) market conditions have also forced investors to look into new channels for investments providing high yields with higher risks, which have not been available from traditional sources of finance, such as banks balance sheet financing or capital markets in general. Both loan and investment-based crowdfunding include many opportunities for investors looking for investments with different return-to-risk ratios. From the investors’ perspective, crowdfunding also offers a new way to diversify investments and seek higher than average profits with a higher risk profile compared to more general investment products available in the market. However, the several hundred years old banking institution is unlikely to vanish any time soon. On the contrary, there is strong indication that some leading business banks have established successful partnerships with crowdfunding platforms and other fintech companies (Nordea 2018; BBVA 2019; Deloitte 2019).

## What Next?

The Fourth Industrial Revolution is underway. According to Statista—a German online portal for statistics, which makes data collected by market and opinion research institutes and data derived from the economic sector and official statistics available—there were about 26 billion devices connected to the internet around the world in 2019. The total installed base of internet connected devices is projected to amount to 75.44 billion worldwide by 2025, a fivefold increase in ten years. The internet of things, enabled by the already ubiquitous internet technology, seems to be the next major step in delivering internet’s promise of making the world a connected place (Statista 2019). Currently it seems that artificial intelligence, machine learning, and the internet of things will have the most effect on the financial sector (Deloitte 2019). Digitalization is currently shaping the financial market sector with a force that has not been experienced in this scale before (Chambers and Dimson 2016). The disruption we are currently witnessing means a development during which many existing policies might be abandoned, and new ones adopted within a relatively short term. This has become even more evident after the outbreak of COVID-19 virus and how it has forced governments, institutions and companies to adopt to new and digitalized ways of working.

The financial market has, throughout its history, experienced tremendous economic and functional breakthroughs and changes, but basic operating models have remained largely unchanged, unlike, for example in industrial and service sectors (Atack and Neal 2009; Chambers and Dimson 2016). However, digitalization and technological advancement have significantly changed people’s behaviour since the beginning of the twenty-first century. The virtual world has entered into all aspects of human life, and modern devices (such as tablets, mobile phones, smart watches, etc.) and applications (such as Facebook, Instagram, WhatsApp, WeChat, Skype, different e-mail applications, etc.) allow people to be continuously reached and contacted. This has given people many new opportunities to improve their living conditions and use of time, but, at the same time, mixed and overlapped time between work and leisure. In parallel, social interaction is increasingly moving into the internet and its complex social media networks (Joinson et al. 2009; Kallio and Vuola 2018).

Not surprisingly, in the same context, people’s expectations towards (financial) service providers have changed. This naturally influences financial markets, which are not a separate fort from the rest of society. People’s expectations as consumers require ongoing development work from the financial markets so that financial market participants are better equipped to meet people’s ever-growing expectations. Nascent technology creates numerous new business opportunities in all business sectors.^6^ In the financial markets of the near future, it is likely that besides existing incumbent market operators, such as banks, also big technology companies like Amazon, Facebook (especially with its proposed cryptocurrency project Libra), Apple, Google, Tencent, or Alibaba, who are already integrating payment services on a large scale to their own services, will take a big share of the markets (Deloitte 2019).

Advances in technology seem to ensure that internet, and other sharing networks, will become more significant and take a larger part of our living environment, which will also inevitably change the financial markets as well, while enabling new service concepts and forms of financing (Morel et al. 2018). This poses challenges for current financial market participants, especially for banks (Deloitte 2019) but also creates a correspondingly high potential to newcomers (disruptors), investors , and companies seeking finance. Lawmakers and market supervisors are facing challenging times, though it is essential to keep in mind that the biggest and often most amazing things happen in a period of big breakthroughs or changes that can at this stage only be expected to accelerate through technological development. With these developments, the role of central banks may be changing rapidly. For instance, ECB is examining whether to develop a digital currency as an alternative to cash (Financial Times 2019). To further support and derive from the project, a body of six central banks (the Bank of England, Bank of Canada, BOE, the Bank of Japan, the European Central Bank, the Riksbank, and the Swiss National Bank, along with the Bank for International Settlements) have been set in order to “share experience as they assess the potential cases for central bank digital currency in their home jurisdictions” (Bloomberg 2020).

Fintech refers to those technological innovations in the field of financial services that may lead to new business models, applications, processes, or products and have a significant ancillary effect on financial markets and institutions in the way financial services are provided. The history of the financial market is full of financial innovations, but the importance of these innovations has grown and market transformation accelerated by technological advances (European Commission 2018a). Accordingly, the market is, at an accelerating pace, deploying various fintech solutions that leverage digital identification, mobile applications, cloud services, big data analysis, artificial intelligence, blockchain, and distributed ledger technologies. New technologies are changing the financial industry and the way consumers and businesses buy services. This creates opportunities for fintech-based solutions that improve access to finance and financial inclusion of digitally networked consumers (PWC 2017).

Today, crowdfunding is used to finance business growth at an accelerated pace. Until recently, it has generally been considered to be appropriate during the seed and growth stages for start-ups and especially small businesses (Spacetec 2014) involving financing from non-professional investors often reaching sums between ten thousand euros to a few million euros. In the financial markets, crowdfunding is typically seen as a high-risk mezzanine as well as debt or equity financing. However, the paradigm might be shifting. The crowdfunding market has already seen institutional interest, which may further accelerate growth of this form of financing (Ziegler et al. 2019). This trend is partly supported by regulation making crowdfunding part of regulated financial markets, as well as governments’ continuous will to impose ever-stricter regulation to existing financial market players.

There may well be an underlying risk that the crowds will be pushed back in the most successful platforms, which are able to show long-term track record especially in debt crowdfunding. If this happens, the credit rating models of platforms would have been battle-tested by the non-professional crowds, but eventually the professional investors will come and harvest the fruit. In addition, deepening deflation in the financial markets seems to push more and more institutional investors to alternative finance in order to pursue profits, which are less available from traditional sources. However, recent research has shown that institutionalization across all crowdfunding model types has actually decreased between 2016 and 2017. This includes funding from pension funds, mutual funds, asset management firms, and banks (Ziegler et al. 2019).

## Conclusion

The size of the crowdfunding market, and hence the importance of this form of financing, has grown rapidly (European Commission 2016) and continues to grow based on recent studies (Ziegler et al. 2019). Crowdfunding transactions taking place digitally on different technological platforms via the internet is a concrete demonstration of how digitalization and business models applying new technological solutions can influence access to finance. Subsequently, such solutions also channel and allocate the limited resources of society to benefit a larger pool of companies, investors , and consumers, and hence supporting the society as a whole.

Nevertheless, crowdfunding is not immune to risks, immorality, opportunism, and moral hazard, which have been witnessed in the financial sector from the start. Here, although the systemic risk may be quite low for the economy as a whole, it is for the benefit of all stakeholders in crowdfunding that some level of governmental control is being exercised. So far, it may be fair to argue that there are no crowdfunding platforms that are “too big to fail”. The business model of crowdfunding involves the ability to seek instant profits from and at the expense of investors, for example, by loosening the service platform’s customer selection. Here, it may be argued that less-informed investors may take risks which better informed investors may not. This risk is highlighted by a fact that crowdfunding platform operators are often start-ups themselves struggling with adequate cash flows and may be pressed to onboard campaigns and investors less selectively. This argument defends reasonable minimum capital requirements.

In the end, the markets naturally repair themselves when investors start to avoid those crowdfunding platforms, which price the risks of their projects and operations poorly or indifferently. For a young and developing industry, the market-based correction mechanism may not necessarily be sufficient, because the industry’s overall reputation can significantly weaken already from one bigger moral gambling case. This is true especially in the current situation where advanced self-regulation of the industry has not yet formed, but competition on market shares and customers has constantly been growing and tightening. The biggest risk for crowdfunding industry and its long-term success would seem to be the industry itself if it does not take these signs of danger seriously enough.

The history of the financial markets is full of innovations, starting from the invention of money and using it as a medium of exchange, the exit from the gold-denominated currency system, and all the way to the increasing popularity of online payment systems. Crowdfunding as a form of financing is part of this series of innovations in the general history of financial markets. Crowdfunding has in quite short period acquired a small but significant position in the international financial markets making it important and accessible funding channel especially for start-ups and SMEs. It can also be stated that crowdfunding has democratized the process of commercialization and financing by making investing in start-ups more widespread and easier to access for all people, instead of being accessible to only high-net-worth individuals, business angels, or venture funds (Ziegler et al. 2019). This has also given new opportunities for companies seeking financing and diversified the functioning of existing financial markets.

The evolution of financial markets or corporate finance naturally will not end in crowdfunding. For example, blockchain technology can, if sufficiently advanced, enable completely new business models that can challenge, when scaled adequately enough, traditional corporate finance as well as crowdfunding as we know it today. Blockchain technology, like other fintech innovations, can have a significant impact on the development of financial markets in the future (Deloitte 2019). This also relates to cyber security and data privacy in general, which are issues that need to be addressed globally in order to capitalize on the benefits of digitalization not just for the good of financial markets, but for the society as a whole (European Commission 2018a). The big challenge for the regulators in this rapidly changing financial environment is to maintain an attitude of “mend it, don’t end it”.

In the future, it is necessary for researchers to further study the historical evolution and development of crowdfunding markets in the wider context of financial markets. The relevance of crowdfunding as a new form of financing to market participants (i.e. investors, companies, and established operators like banks) would be worthwhile researching. This is especially true in the current exceptional financial market environment, which is characterized by zero or even negative interest rates, as well as continuous liquidity injections by central banks and government-led projects or initiatives (especially in Europe), such as the European Fund for Strategic Investments and its local counterparts. An alternative historical approach may be comparisons between the development of crowdfunding and other innovations in financial markets, highlighting common and different drivers and barriers to such developments, and the actors behind them. There is also a need for more (historical) study on both the positive and negative implications of financial innovations (including crowdfunding), the determinants of risk taking by institutional and individual investors, the governance problems (including conflict of interest between different stakeholders), and the causes of volatility in financial markets in relation to emergence of fintech. All these issues have practical implications to the success and implementation speed of new financial innovations to practice and everyday service offering and use by individual banks, companies, and households.

Also increasing regulatory burden, which has mostly fallen on the shoulders of established financial institutions like banks, might distort the functioning of financial markets even further and create more concerns among investors and in the public, which can have unprecedented effects to the financial markets of today. Big shifts in current paradigm in the financial markets can make crowdfunding more attractive to institutional investors in the future. The relationship between institutional investors and crowdfunding platforms is a particularly interesting research opportunity, as it may have profound effects on industry development, and the extent to which it will remain loyal to its grass-root ideals.

Further, more research is needed on the effects on and implications of the crowdfunding industry on systemic risk especially if the growth of the industry continues as strong as it has to date. It will also be interesting to follow how quickly and agile crowdfunding platforms will adapt innovations of fintech (blockchain, AI, cloud computing, etc.) into their everyday operations compared to, for example, banks, as well as what effects will that have on the future position and service portfolios of platforms and banks respectively. In growing markets, there also seems to be an increasing pressure for consolidation of the crowdfunding platforms as well as expansion of their current product lines and ability to adapt to new and more scalable business models (e.g. setting up alternative investment funds in order to ensure steadier cash flow as well as expanding from solely lending-based crowdfunding to cover other crowdfunding forms). In addition, it is interesting to study the increasing syndication and cooperation activities between traditional banks and crowdfunding platforms.

In conclusion, we are living in interesting times of constantly evolving financial markets. In order to be able to predict future trends and directions, we must understand the past and derive from the teachings of history; and in this particular case-financial history. In order to understand history, it is essential not to highlight only similarities between historical episodes such as the Great Depression of 1929 and the subprime crisis of 2007 but also differences.^7^ Such an approach shows us that history does not always have such a conclusive predictive power than we would probably like it to have (Chambers and Dimson 2016). However, history has always provided invaluable guidance to those willing to learn from events, and especially mistakes, of the past. At the finalizing phase of this chapter we are witnessing an outbreak of COVID-19 virus that hammers the global economy at forces rarely seen before. The outcomes of such crises are hard to predict. We might be entering into a beginning of a new era of disintegration in EU and rising levels of nationalisation. On the other hand, the solidarity may even strenghten among EU states, and the level of global co-operation and transparency might increase and improve.

From practical standpoint, it is useful to contextualize crowdfunding—a modern and digitalized form of financing—as part of financial markets, its rules, and mechanics. In order to achieve such a goal, it is essential to understand fluctuations between economic cycles driven by historical, economic, and political processes. Crowdfunding and fintech in general will definitely offer many interesting research topics for researchers in the financial markets for years to come.

## Footnotes

1 Although the economic history of the past 300 years has shown many instances of financial crises and bank failures, their effects on asset values and on the real economy have been quite varied. Much depends on how the authorities react to these events. Although we understand from history the factors that lead to and exacerbate crises, the attempts to make such crises less frequent and less virulent have been largely misguided (Chambers and Dimson 2016, p. xvi).

2 “Economic bubble” means a situation in the economy where a price of a trading object (e.g. a stock or other commodity) differs from the balanced price defined by the fundamentals of the market (based on availability, valuation, supply, etc.). The bubble may form, for example, to the prices of stocks or house prices (Chambers and Dimson 2016, pp. 149–168 and 174–175). One of the traditional and frequently used examples of “economic bubble” is the so-called tulip mania, which was an investment bubble in the Netherlands between 1634 and 1637, where prices of tulip bulbs entering the markets most recently rose to record levels and eventually collapsed. The highest price of tulips was in 1637. Tulip mania is considered one of the first economic bubbles in the modern financial markets.

3 Economic cycles affect, among others, (i) the number of loan transactions, (ii) the size of the loan facilities, (iii) pricing for the loans, (iv) default rates, and (v) institutional demand for secondary trading. A buoyant economy with low default rates encourages lenders to commit to large loans and more frequently, whereas a contracting economy usually results in a more cautious approach in the number of transactions and the terms on which the loans are made. A deteriorating economy with high default rates spurs the secondary market in distressed loans (Mugasha 2007, p. 6).

4 Jonathan Swift is better known as an author of a well-known prose satire; Gulliver’s Travels, or Travels into Several Remote Nations of the World. In Four Parts. By Lemuel Gulliver, First a Surgeon, and then a Captain of Several Ships.

5 US banks, compared to their European counterparts, are ahead on multiple measures. Aggressive policy interventions and forceful regulations helped propel US banks to health more quickly. And more recently, favourable GDP growth, tax cuts, and rising rates have further bolstered the state of the industry. Total assets in the US reached a peak of $17.5 trillion. Capital levels are up as well, with average tier 1 capital ratio standing at 13.14%. Return on equity (ROE) for the industry is at a post-crisis high of 11.83%. Efficiency ratios also are at their best. Similarly, on other metrics, such as non-performing loans and number of failed institutions, the US banking industry is robust (Deloitte 2019, p. 1).

6 For example: (i) video rentals (Blockbusters) have gone online (Netflix, Viaplay, HBO), (ii) instead of CDs, music is listened online via web streaming services (Spotify), (iii) instead of travel agencies, most trips and accommodations are booked directly through internet platforms (e-aggregators) (Trivago, Ebookers), and (iv) instead of visiting branches, banking is handled through online banking channels or, increasingly, via banking applications on mobile phones.

7 For example, complex and highly automatized/digitalized structured financial products—non-existent during crisis of the 1930s—were vastly used in global financial markets prior to the subprime crisis. The use of as well as lack of understanding related to these products has been identified as one of the major contributing factors to the subprime crisis (Chambers and Dimson 2016, pp. 272–276).
